# High-risk coronary plaque in SLE: low-attenuation non-calcified coronary plaque and positive remodelling index

**DOI:** 10.1136/lupus-2020-000409

**Published:** 2020-07-28

**Authors:** George Stojan, Jessica Li, Matthew Budoff, Armin Arbab-Zadeh, Michelle A Petri

**Affiliations:** 1 Rheumatology, Johns Hopkins University, Baltimore, Maryland, USA; 2 Cardiology, David Geffen School of Medicine, Los Angeles, California, USA; 3 Cardiology, UCLA, Los Angeles, California, USA; 4 Cardiology, Johns Hopkins University, Baltimore, Maryland, USA; 5 Division of Rheumatology, Johns Hopkins University School of Medicine, Baltimore, Maryland, USA

**Keywords:** lupus erythematosus, systemic, atherosclerosis, cardiovascular diseases

## Abstract

**Background:**

Positive remodelling index and presence of low-attenuation non-calcified plaque (LANCP) are characteristic vessel changes in unstable coronary plaques. We sought to characterise these high-risk plaque features in patients with systemic lupus erythematosus (SLE) and to compare them with controls.

**Methods:**

A total of 72 patients who satisfied the SLICC classification criteria for SLE had coronary CT angiography (CCTA) studies, 30 of which had follow-up CCTA, as screening for occult coronary atherosclerotic disease in asymptomatic individuals. A total of 100 consecutive controls with no known history of lupus, heart disease or revascularisation who had two coronary CT angiograms at least 1 year apart were included in the study. These were asymptomatic patients referred by their primary physicians for screening of coronary artery disease and the screening interval was decided by the primary physicians. The methodology for image acquisition was identical.

**Results:**

LANCP burden at baseline was significantly greater in patients with SLE compared with controls. LANCP volume was significantly greater in patients over 60 years of age (p<0.05) and in those with current prednisone dose >10 mg/day. LANCP burden remained stable over follow-up. There were no significant differences in remodelling index compared with controls.

**Conclusion:**

This is the first study describing high-risk CCTA features of coronary plaque in patients with SLE. Both LANCP and positive remodelling are common in SLE. These characteristic vessel changes may identify patients with SLE at increased risk of cardiovascular events and those in need for more frequent cardiac monitoring.

## Background

Low-attenuation non-calcified plaque (LANCP) and positive remodelling index are characteristic vessel changes in unstable coronary plaques.^1^

LANCP (<30 Hounsfield Units (HU)) contain necrotic cores that are characterised by endothelial dysfunction, oxidative stress and inflammation.^2^ They have been shown to be a better predictor of future cardiovascular events compared with traditional cardiovascular risk factors in the general population.^3^

Coronary arterial remodelling describes changes of vessel size at the site of atherosclerotic lesions.^4^ Positive remodelling (expansion) of early lesions maintains lumen size despite plaque accumulation.^5^ This explains why these lesions might pass undetected by conventional angiography. Coronary artery plaques with positive remodelling have a higher lipid content and macrophage count, both markers of plaque vulnerability.^6^ Plaque rupture is often apparent at sites with only modest luminal stenoses but marked positive remodelling.^7^

When low-attenuation and positive remodelling coexist, the risk for future cardiovascular events is 11–22-fold higher than patients without these characteristics.^1 8^

Accelerated atherosclerosis leading to premature coronary artery disease remains the major cause of late death in systemic lupus erythematosus (SLE).^9^ We sought to characterise LANCP and positive remodelling index in patients with SLE.

## Methods

As previously described,^10^ the Hopkins Lupus Cohort is a prospective cohort study of predictors of lupus flare, atherosclerosis and health status in SLE. The study cohort includes all patients at the Hopkins Lupus Center who have a clinical diagnosis of SLE and give informed consent to participate in the study. Subjects enrolled in the cohort are followed quarterly or more frequently if clinically necessary. The clinical features, laboratory testing and damage accrual data are recorded at the time of entry into the cohort and are updated at subsequent visits. The Hopkins Lupus Cohort has been approved by the Johns Hopkins University School of Medicine Institutional Review Board and complies with the Health Insurance Portability and Accountability Act. All patients gave written informed consent.

A total of 72 patients who satisfied the Systemic Lupus International Collaborating Clinics (SLICC) classification criteria for SLE^11^ had coronary CT angiography (CCTA) studies, 30 of which had follow-up CCTA (mean=3.77 years, SD=0.94 years), for screening for occult coronary atherosclerotic disease in asymptomatic individuals. Follow-up CCTA was part of the study protocol for all patients but due to expiration of funding, follow-up CCTA was completed in 30 patients. Exclusion criteria included a serum creatinine level ≥1.2 mg/dL, allergy to contrast material, pregnancy or history of angina, myocardial infarction or stroke attributable to atherosclerotic disease. CT images were acquired using a 320×0.5 mm-detector row CT system (Aquilion ONE, Canon Medical Systems, Otawara, Japan). Patient preparation included oral (75–150 mg) metoprolol. Fifty to seventy millilitres of iodinated contrast (Iopamidol 370 or iohexol 350 mg iodine/mL) was injected intravenously at 4.0–6.0 mL/s for prospectively ECG-triggered acquisitions (typically 75% of the R-R interval). If coronary calcification was evident on angiographic images, a non-contrast CT acquisition was added to quantify calcium for the Agatston score. CT angiographic data were reconstructed to generate 0.5 mm slice thickness images with a 0.25 mm increment using both a standard (FC43) and a sharp (FC05) convolution kernel. Reconstructed images were transferred to a dedicated workstation for further analysis (Vitrea FX V.3.0 workstation, Vital Images, Minnetonka, Minnesota, USA). Of 52 patients with SLE not on statins at baseline, 19 patients were started on statins after the first CCTA.

A total of 100 consecutive controls with no known history of lupus, heart disease or revascularisation^12^ who had two coronary CT angiograms at least 1 year apart (mean=1.11 years, SD=0.25 years) were included in the study. These were asymptomatic patients referred by their primary physicians for screening of coronary artery disease and the screening interval was decided by the primary physicians. The methodology for image acquisition was similar^12 13^ and three-dimensional image analysis was performed on identical workstation (Vitrea FX V.3.0 workstation, Vital Images, Minnetonka, Minnesota, USA). Of 28 controls not on statins at baseline, 6 were started on statins after the first CCTA. Statin information was only available at the time of the baseline scan or the follow-up, so we could not assess duration of prior statin use for the controls.

All images were interpreted by a physician, blinded to any clinical information, with extensive experience (level III certified) in the interpretation of CCTA. The left anterior descending coronary artery, left circumflex coronary artery and right coronary artery were divided into proximal, mid and distal segments, respectively, according to standard segmentation guidelines.^14^ The left main coronary artery was evaluated separately, resulting in 10 segments evaluated in total. Each segment was assessed for the presence and severity of atherosclerotic plaques on a semiquantitative scale. Plaque characteristics, including remodelling, were noted for each segment. Spotty calcification and the napkin-ring sign were not analysed.

Each non-calcified plaque (NCP) detected within the vessel wall (left anterior descending artery, right coronary artery and left circumflex) was evaluated with the minimum CT density. A plaque density <30 HU was considered low attenuation.^15^

Coronary plaque area was measured by manual tracing for the difference between the area within the external elastic membrane and the area of the vessel lumen at the site of maximal luminal narrowing as observed on a cross-sectional CCTA. A LANCP was defined as a low-attenuation (<30 HU) mass >1 mm^2^ in size, located within the vessel wall and clearly distinguishable from the contrast-enhanced coronary lumen and the surrounding pericardial tissue.

Lesions with a remodelling ratio >1.05 were considered to have positive remodelling, and those in which remodelling was <0.95 were considered to have negative remodelling.^16^ Lesions with a remodelling ratio of 0.95–1.05 were classified as having no remodelling.^16^

### Statistical analysis

χ² test was used to evaluate baseline characteristics between lupus patients and controls. Paired t-test or Wilcoxon signed ranks test was used to compare plaque volume between baseline and follow-up for each cohort. A linear regression model was used to compare the baseline plaque volumes between patients with SLE and controls adjusting for the factors that were found to be significant from the baseline demographics, namely age, sex, ethnicity, diabetes and statin use. In both cohorts, age was a continuous variable. Ethnicity categories were defined as African American, Caucasian and others.

Analysis of variance was used to evaluate the association between LANCP and demographic and clinical variables including sex, race, age at baseline, ever smoking (from history file), diabetes, hypertension, hyperlipidaemia, obesity, lupus anticoagulant, anticardiolipin, anti-beta 2 glycoprotein, hydroxychloroquine and immunosuppressant use, anti-dsDNA, low C3, low C4, antihypertensive and statin use.

## Results

Table 1 describes the baseline characteristics of the patients with SLE and controls. At the time of their baseline CCTA, a higher proportion of controls were male, older, diabetic and on a statin. Also, there were fewer African Americans among controls. Characteristics of patients with SLE are detailed in table 2. The average disease duration at the time of the first CCTA was 12.0±1.9 years.

**Table 1:** Baseline demographic characteristics of patients with SLE and controls

	Patients with SLE (n=72)	Controls (n=100)	P value
Age at baseline, mean (SD)	51.1 (SD=11.4)	66.3 (SD=9.6)	
Age at baseline categories			<0.0001
* *<45	27 (37.5%)	2 (2%)	
* *45–<60	37 (51.4%)	25 (25%)	
* *≥60	8 (11.1%)	73 (73%)	
Sex			<0.0001
Female	61 (84.7%)	24 (24%)	
Male	11 (15.3%)	76 (76%)	
Ethnicities			0.0003
African American	17 (23.6%)	5 (5.3%)	
Caucasian	51 (70.8%)	67 (71.3%)	
Other	4 (5.6%)	19 (20.3%)	
Ever smoking			0.8820
Yes	24 (33.3%)	31 (34.4%)	
No	48 (66.7%)	59 (65.6%)	
Hypertension			0.9590
Yes	47 (65.3%)	61 (64.9%)	
No	25 (34.7%)	33 (35.1%)	
Diabetes			0.0013
Yes	2 (2.8%)	18 (19.1%)	
No	70 (97.2%)	76 (80.9%)	
Statin use			<0.0001
Yes	20 (27.8%)	63 (68.5%)	
No	52 (72.2%)	29 (31.5%)	

Sample sizes for each variable do not always add up to the total due to missing information in some patients.

SLE, systemic lupus erythematosus.

**Table 2:** Baseline characteristics of patients with SLE

Characteristics of patients with SLE (n=72)	
	N (%) or mean±SD
Disease duration*	12.0±1.9
History of	
Malar rash	27 (37.5%)
Discoid	9 (12.5%)
Mouth ulcers	41 (56.9%)
Alopecia	39 (54.2%)
Arthritis	57 (79.2%)
Serositis	36 (50.0%)
Proteinuria	27 (37.5%)
Seizure	2 (2.8%)
Haemolytic anaemia	6 (8.3%)
Leucopaenia	37 (51.4%)
Thrombocytopaenia	15 (20.8%)
ANA	70 (97.2%)
Anti-dsDNA	38 (52.8%)
Lupus anticoagulant	24 (33.3%)
Anticardiolipin	51 (71.8%)
Anti-B2 glycoprotein	24 (33.3%)
Low C3	46 (63.9%)
Low C4	35 (48.6%)
Direct Coomb’s test	12 (16.7%)
At baseline CTA	
SELENA-SLEDAI	1.7±2.1
PGA	0.6±0.7
Hydroxychloroquine use	63 (87.5%)
Immunosuppressant use	26 (36.1%)
Prednisone use	15 (20.8%)
Statin use	20 (27.8%)
Antihypertensive use	44 (61.1%)

*Disease duration at the time of baseline CTA.

CTA, CT angiography; PGA, physician global assessment; SELENA-SLEDAI, Safety of Estrogens in Lupus National Assessment-Systemic Lupus Erythematosus Disease Activity Index; SLE, systemic lupus erythematosus.

Table 3 provides mean total plaque and calcified plaque volume at baseline in patients with lupus and controls. The significantly higher burden of calcified plaque in controls is expected due to the older age and predominantly male population, but the total plaque and NCP burden was significantly higher in the SLE group, even after adjusting for age, sex, ethnicity, diabetes and statin use.

**Table 3:** Plaque volumes (in mm^3^) between study groups at baseline

	Unadjusted associations			Adjusted associations*		
	SLE group (n=72) Mean estimate (95% CI)	Control group (n=100) Mean estimate (95% CI)	P value	SLE group (n=72) Mean estimate (95% CI)	Control group (n=100) Mean estimate (95% CI)	P value
Total plaque volume	1483.5 (1381.7 to 1585.3)	477.4 (391.0 to 563.9)	<0.0001	1695.3 (1521.6 to 1868.9)	359.6 (211.7 to 507.6)	<0.0001
Calcified plaque volume	9.8 (−49.6 to 69.1)	280.6 (230.3 to 330.9)	<0.0001	64.9 (−45.8 to 175.6)	224.1 (129.9 to 318.4)	0.0264
NCP volume	1473.8 (1392.2 to 1555.3)	196.8 (127.8 to 266.0)	<0.0001	1630.4 (1494.1 to 1766.7)	135.5 (19.4 to 251.6)	<0.0001

*Adjusted for sex, ethnicity, diabetes, statin use at baseline and age at baseline.

NCP, non-calcified plaque; SLE, systemic lupus erythematosus.

Table 4 displays the mean of the total LANCP volume in mm^3^ at baseline in patients with SLE and controls. Despite the predominance of elderly males among controls, patients with SLE had a significantly higher burden of LANCP (p<0.001). Middle-aged women with SLE (age 45–59) had a substantially greater LANCP burden compared with controls although there was no statistically significant difference in patients aged 60 or greater, possibly due to the small number of women with lupus in this group. Importantly, the control group included no women younger than 45 and the number of male patients with SLE was small.

**Table 4:** Mean (SD) of low-attenuation non-calcified plaque at baseline, in each cohort, by age and sex

Sex	Age	Low-attenuation non-calcified plaque				
		SLE		Controls		P value
		n	Mean (SD)	n	Mean (SD)	
All	All	66	457.92 (207.53)	100	42.19 (52.30)	<0.0001
Female	<45	19	393.63 (119.18)	0	– (–)	–
	45–59	33	450.52 (150.37)	3	53.37 (31.6)	<0.0001
	60+	5	695.2 (570.58)	21	21.58 (25.59)	0.0576
Male	<45	3	412.33 (216.58)	2	51.15 (72.34)	0.1176
	45–59	5	516.4 (76.83)	22	46.84 (57.8)	<0.0001
	60+	1	582 (–)	52	47.56 (57.54)	–

SLE, systemic lupus erythematosus.

Table 5 provides mean remodelling index at baseline in patients with lupus and controls. No significant differences between the two groups were found but the numbers were small. Among patients with lupus, only women>60 and men>45 had a mean positive remodelling index while all other age groups had mean negative remodelling.

**Table 5:** Mean (SD) of remodelling index at baseline in each cohort by age and sex

Sex	Age	Remodelling index				
		SLE		Control		P value
		n	Mean (SD)	n	Mean (SD)	
All	All	66	0.96 (0.69)	91	0.97 (0.28)	0.8412
Female	<45	19	0.89 (0.55)	0	- (-)	–
	45–59	33	0.89 (0.78)	3	0.85 (0.09)	0.8147
	60+	5	1.30 (0.59)	20	0.85 (0.33)	0.1663
Male	<45	3	0.83 (0.42)	2	1.28 (0.47)	0.3845
	45–59	5	1.34 (0.75)	18	0.95 (0.29)	0.3038
	60+	1	1.26 (–)	47	1.03 (0.24)	–

SLE, systemic lupus erythematosus.

Table 6 details the changes in LANCP and remodelling index between the two CCTA in lupus and controls, respectively. No significant change was seen in LANCP volume in SLE, while a significant regression in LANCP volume was seen in the control group. No significant changes were seen over time in remodelling index in either group.

**Table 6:** Volume progression/regression (mm^3^) within each cohort

	SLE			Control		
	n	Mean diff (SD)	P value	n	Mean diff (SD)	P value
Change in total NCP plaque	70	6.27 (322.46)	0.8712	100	−32.84 (110.67)	**<0.0001**
Change in LANCP plaque	27	−13.56 (93.29)	0.4570	100	−6.90 (27.62)	**0.0002**
Change in remodelling index	30	0.09 (0.87)	0.6013	83	−0.03 (0.38)	0.4062

LANCP, low-attenuation non-calcified plaque; SLE, systemic lupus erythematosus.

Table 7 shows the association between patient and disease-specific variables with LANCP. LANCP was associated with age (p<0.05) and current prednisone dose >10 mg/day. There were only five cardiovascular events in the studied group so the association of LANCP and positive remodelling index could not be assessed.

**Table 7:** Mean (SD) of LANCP volume (mm^3^) in SLE subgroups

Demographic and clinical variables	Subgroup	LANCP mean (SD)	P value
Sex	Female (n=57)	453 (217)	0.6325
	Male (n=9)	489 (136	
Race	White (n=43)	459 (223)	0.3823
	Black (n=19)	483 (180)	
	Other (n=4)	324 (107)	
Age at baseline	<45 (n=22)	396 (129)	0.0115
	45–59 (n=38)	459 (144)	
	60+ (n=6)	676 (512)	
History of smoking	Ever (n=20)	413 (147)	0.2482
	Never (n=46)	478 (228)	
History of lupus anticoagulant	Yes (n=23)	504 (291)	0.1880
	No (n=43)	433 (143)	
History of anticardiolipin	Yes (n=46)	450 (236)	0.6527
	No (n=20)	476 (122)	
Current cholesterol 200+	Yes (n=12)	465 (218)	0.6042
	No (n=53)	430 (168)	
Current BMI	<25(n=25)	430 (284)	0.3007
	25–30 (n=21)	434 (118)	
	>30 (n=20)	518 (158)	
Current anti-dsDNA	Yes (n=13)	517 (375)	0.2558
	No (n=53)	443 (143)	
Current low complement	Yes (n=16)	469 (354)	0.8116
	No (n=50)	454 (137)	
Current hydroxychloroquine use	Yes (n=56)	466 (215)	0.4677
	No (n=10)	414 (158)	
Current prednisone use	<10 mg/day (n=62)	436 (143)	0.0004
	≥10 mg/day (n=4)	800 (591)	
Current SLEDAI	≤3 (n=48)	445 (135)	0.4198
	>3 (n=18)	492 (335)	
Hypertension or antihypertensive use	Yes (n=46)	482 (236)	0.1629
	No (n=20)	404 (107)	

BMI, body mass index; LANCP, low-attenuation non-calcified plaque; SLE, systemic lupus erythematosus.

## Discussion

CCTA has emerged as the best non-invasive imaging modality in terms of assessing both lumen area and plaque composition.^17^ Absence of atherosclerosis on CCTA has a negative prognostic value approaching 100%, even in prolonged follow‐up.^18^ Pundziute *et al*^19^ demonstrated that CCTA provides independent prognostic information over baseline clinical risk factors in patients with known and suspected Coronary Artery Disease (CAD). The type of plaque identified on CCTA has important predictive value with mortality incrementally increasing from calcified plaque (1.4%) to partially calcified plaque (3.3%) to NCP (9.6%).^20^

Regarding vulnerable plaques, CCTA can assess the presence, size and thickness of the necrotic core, by grading tissue in HU; plaques with large cores will cause less attenuation and thus have lower unit values.^21^ Specific high‐risk plaque criteria have been developed, such as positive remodelling (remodelling index ≥1.1^22^) and (very) low (given that the threshold for a≥10% necrotic core is 41 HU) attenuation NCP (<30 HU).^17^

A recent CCTA study, with a follow‐up of almost 100 months,^23^ confirmed that, in non-obstructive disease, positive remodelling index, increased plaque burden and LANCP were all independently associated with cardiac events. Presence of both positive remodelling index and LANCP yielded a more than 22% probability of acute coronary syndrome (ACS) over an average follow‐up of 27 months, as compared with <0.5% probability, should both features be absent.^1^

In a previous analysis of presence or absence of NCP in SLE, we found that 54% of patients with SLE had NCP,^24^ that 53% of patients who had no coronary calcification had NCP^25^ and that NCP was present more often in those with higher disease activity, current immunosuppressive use and current nonsteroidal anti-inflammatory drugs (NSAID) use,^24^ although a subsequent quantitative analysis failed to show an association with measures of SLE disease activity.^25^ Patient age, current methotrexate use, history of anti-dsDNA positivity and obesity were associated with the presence of non-calcified coronary plaque, in the multivariate model.^25^ We thus decided to assess for the first time high-risk plaque characteristics in patients with SLE, namely LANCP and positive remodelling index, and compare their burden with a control population.

Total plaque and NCP burden were significantly higher among patients with SLE even after adjusting for age, sex, ethnicity, diabetes and statin use. LANCP burden at baseline was also significantly higher in patients with SLE compared with controls, even though our control group consisted mostly of elderly men. LANCP volume was significantly higher in patients with SLE over 60 years of age (p<0.01) and in those with current prednisone dose >10 mg/day. There was no association with any measure of SLE disease activity, antiphospholipid antibodies, smoking, hypertension, hypercholesterolemia or immunosuppressant use. The number of cardiovascular events was small (n=5), so the association with low-attenuation plaque and positive remodelling index could not be assessed.

LANCP burden remained stable over follow-up in contrast to controls, where there was significant regression of LANCP over time. Statin use had no association with LANCP regression over time in patients with SLE or in controls.

While a mean negative remodelling index was seen in women<60 years of age and men<45 years of age, the mean remodelling index in women over 60 years of age was 1.3 (SD=0.59) and in men>45 years of age, it was 1.34 (SD=0.75). There were no significant differences compared with controls and no associations with any demographic characteristic, traditional cardiovascular risk factor or SLE-specific risk factor. Remodelling index remained stable over time in both patients with SLE and controls.

### Limitations

We acknowledge several study limitations. Our control group is not matched to the characteristics of the study population which hinders optimal comparison. We used regression to compare the two groups, but some of the cell sizes are extremely small. For instance, among controls, only 2% (2 patients) were less than age 45, with only 5 African Americans (5%) and a relatively low representation for female sex at 24 patients (24%). Among patients with lupus, only 2.8% (two patients) were diabetic. Adverse event rate is very low in our study and associations of plaque findings with patient outcome cannot be assessed. Finally, the value of CT characteristics of coronary atherosclerotic plaque vulnerability remains uncertain. While they have shown promise for prognostic information compared with established predictors, their incremental value may be small.^26^

## Conclusion

This is the first study describing high-risk CCTA features of coronary plaque in patients with SLE. Both LANCP and positive remodelling are common in SLE. Importantly, these characteristic vessel changes seen in unstable coronary plaques persisted over time and were not associated with any disease-specific risk factor. Their presence may identify patients with SLE at highest risk of cardiovascular events and those in need for more frequent cardiac monitoring.
